# Quantification of microRNA in plasma using probe based TaqMan assays: is microRNA purification required?

**DOI:** 10.1186/s13104-019-4301-5

**Published:** 2019-05-10

**Authors:** Helle Glud Binderup, Jonna Skov Madsen, Claus Lohman Brasen, Kim Houlind, Rikke Fredslund Andersen

**Affiliations:** 1 Biochemistry and Immunology, Lillebaelt Hospital, Sygehusvej 1, 6000 Kolding, Denmark; 2 Department of Vascular Surgery, Lillebaelt Hospital, Kolding, Denmark; 3 Department of Regional Health Research, University of Southern Denmark, Kolding, Denmark

**Keywords:** MicroRNA, Direct plasma RT-qPCR, Sample preparation, TaqMan assays

## Abstract

**Objective:**

Circulating microRNAs are promising diagnostics and prognostics biomarkers in a wide variety of diseases. However, there is a critical reproducibility challenge, which in part may be due to preanalytical factors. MicroRNA purification has been identified as the major contributor to the total intra assay variation, thus we found great interest in recent papers describing methods for direct quantification of circulating microRNAs without the purification step. With one exception, all the studies we identified where a direct quantification of circulating microRNAs had been performed were using SYBR Green chemistry. In our laboratory we use platelet-poor plasma and TaqMan assays for microRNA analysis, and thus we investigated whether we could adapt the procedures for the direct reverse transcription described by these studies to be used with our TaqMan assays.

**Results:**

We did not achieve valid results by direct quantification of selected microRNAs (miR-92a, miR-16 and miR-126) in platelet-poor plasma using TaqMan assays.

**Electronic supplementary material:**

The online version of this article (10.1186/s13104-019-4301-5) contains supplementary material, which is available to authorized users.

## Introduction

MicroRNAs have been demonstrated to be involved in virtually every aspect of cell biology, and a large number of papers have been published showing microRNAs as promising diagnostic and prognostic biomarkers in a wide variety of diseases. In many cases, however, subsequent studies failed to reproduce the original findings [1–4]. Many reasons for this reproducibility challenge might play a role, among which some are related to preanalytical and analytical factors: Thus, McDonald et al. found an intra assay variation of up to 0.3 Ct (equal to 1.23 fold or 23%) and estimated that microRNA-purification accounted for 77–92% of this variation [5]. In line with this, our group recently found that plasma preparation and microRNA purification accounted for 64–73% of the total intra-assay variation when quantifying miR-92a, miR-16 and miR-126 in platelet-poor plasma using TaqMan assays and spiking samples with cel-miR-39 as a means of normalization [6]. Therefore, it would be tempting to leave out this purification step in favor of a direct quantification, which might be possible according to promising results reported by other research groups [7–11]. With one exception [8], all the identified papers describing a method for direct quantification of circulating microRNA were using SYBR Green chemistry [7–15]. In our group we use TaqMan assays for microRNA analysis, and since studies comparing SYBR Green and TaqMan assays found that both methods are reliable, but that results obtained by the two methods in some cases are inconsistent, the choice of methodology is important [16, 17]. Furthermore, we have special interest in plasma levels of platelet-derived microRNAs (e.g. miR-92a, miR-16 and miR-126), so to minimize the contamination with microRNAs contained inside the platelets we perform the reverse transcription with microRNA samples purified from platelet-poor plasma. Therefore, with the intention to improve quality and reproducibility of our microRNA analysis, we investigated whether we could adapt the procedures for direct reverse transcription previously described by other studies to be used with our TaqMan assays on platelet-poor plasma.

## Main text

### Methods

Platelet-poor plasma (PPP), EDTA-plasma and serum samples from 10 healthy staff-members were used to investigate various approaches to perform RT-qPCR directly in plasma using TaqMan assays (Applied Biosystems, Foster City, CA). Since the samples were kept anonymized and the purpose of the study fell within the category “Quality control and quality development”, we did not need to notify the Regional Ethical Committee for the region of Southern Denmark (http://en.nvk.dk/how-to-notify/what-to-notify).

PPP was prepared from EDTA whole blood by dual centrifugation; a detailed protocol is available at protocols.io (10.17504/protocols.io.q9edz3e). EDTA-plasma and serum was obtained after a 10 min centrifugation at 2000*g* (room temperature).

In order to deactivate plasma proteins that may interfere with the RT-qPCR, plasma was mixed 1:1 with a denaturing buffer composed of 2.5% Tween-20, 50 mM Tris–HCl and 1 mM EDTA (all from Sigma-Aldrich, Inc., St. Louis, MO, USA) as described in other studies using the RT-qPCR direct approach [7, 8]. Since none of the studies specifies the pH of their denaturing buffer, two versions were used, one in which we did not adjust pH, and one in which pH was adjusted to 8.0, which is the pH that is used for other Tris–EDTA buffers in our laboratory. The plasma and buffer mixtures were either used directly for cDNA synthesis or further processed by e.g. heating and centrifugation. As a means of normalization a volume of Cel-miR-39 (2.75 × 10^−12^ M) (RiboTask, Odense, Denmark) was spiked in during the sample preparation or added to the RT-mixture.

The synthesized cDNA was either used directly or centrifuged before used as template in the qPCR, which in all cases were performed in doublets with 1.3 µL of cDNA in at total reaction volume of 20.3 µL. All analyses were performed using TaqMan assays for miR-92a, miR-126, miR-16 and Cel-miR-39, and in all experiments a purified microRNA sample (from PPP) was included as a positive control.

A detailed description of the microRNA purification kit, reverse transcription kit, TaqMan assays, PCR master mix and thermocycler conditions used is available at protocols.io (10.17504/protocols.io.q9edz3e).

An overview of the different approaches tested is given in Table 1.

**Table 1:** Overview of RT-qPCR approaches tested

Approach	Plasma preparation	RT-reaction	Proceeding of cDNA	References
1	5 µL plasma + 5 µL denaturing buffer Incubation at 75 °C for 5 min, cool on ice Ad 2 µL spike^a^ Centrifugation at 10,000*g* for 10 min at 4 °C	6 µL plasma preparation in a total volume of 15 µL	None	Zhao et al. [9]
2	2.5 µL plasma + 2.5 µL denaturing buffer Ad 1 µL spike^a^	5 µL plasma preparation in a total volume of 15 µL	Centrifugation at 10,000*g* for 10 min	Asaga et al. [7] Zheng et al. [12]
3	2.5 µL plasma + 2.5 µL denaturing buffer Ad RNase inhibitor and RT-primer pool Incubation at 70 °C for 10 min	Ad rest of RT-reaction mixture and 1 µL spike^a^ (total 15 µL)	Centrifugtion at 10,000*g* for 10 min	Liu et al. [8]
4	5 µL plasma + 5 µL denaturing buffer Ad RNase inhibitor and 2 µL spike^a^ Incubation at 70 °C for 10 min, cool on ice Centrifugation at 10,000*g* for 10 min at 4 °C	6 µL plasma preparation in a total volume of 15 µL	None	

Overview of different RT-qPCR approaches tested in order to perform direct plasma analysis of microRNA-levels. The source for inspiration to each test procedure is provided in the last column

^a^Cel-miR-39 (2.75 × 10^−12^ M)

### Results and discussion

Using approach number 1 Cel-miR-39 was first added together with the buffer, which resulted in the spike being undetermined in all samples. Therefore, we decided to add the spike after the incubation step, but the Ct-values still were very high (range: 36–42), possibly due to digestion of the synthetic microRNA by enzymes in the plasma. Ct-values of the target microRNAs were also found to be high (range: 30–42) and several samples were undetermined.

With Approach number 2 all measured Ct-values were > 35, which we considered outside of the measuring range. As with approach number 1, this indicates that the buffer itself is insufficient to deactivate the plasma proteins.

**Fig. 1:**
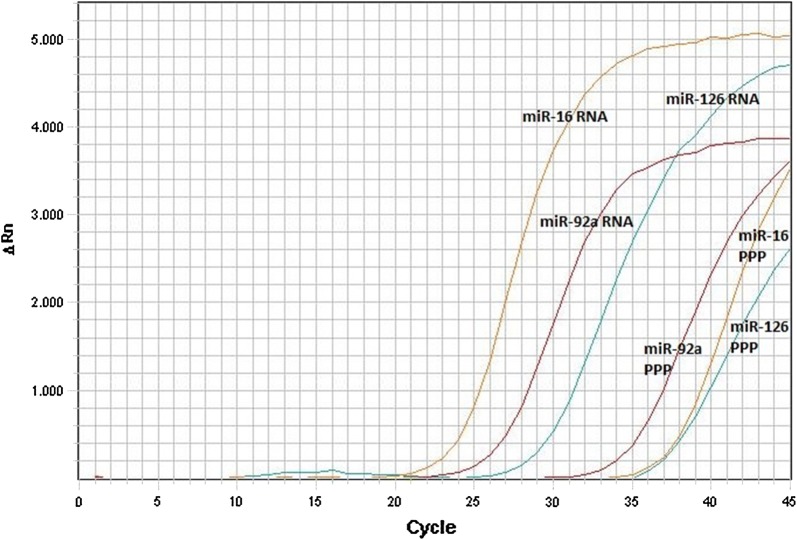
Example of amplification plot for approach number 3. The amplification of miR-16, miR-92a and miR-126 when using platelet-poor plasma (PPP) direct as template for reverse transcription compared to analysis using miRNA purified from the same PPP (RNA). The reverse transcription and qPCR were performed in the same runs

When Approach number 3 was used, the Ct-value for cel-miR-39 was approximately 27 (range: 25–27) in all samples. For the target microRNAs Ct-values were in the range 28–40, and the duplicate measurements differed by 0–2 Ct-values. Compared to approach 1 and 2, this indicates that adding the RNase to the sample-buffer mixture prior to the incubation step, helped to deactivate the plasma proteins. Unexpectedly, for the highly expressed miR-16 Ct-values were > 34 in all samples. An example of the amplification plots obtained for miR-92a, miR-16 and miR-126 when using a PPP sample directly as template for reverse transcription and when analysis was performed with purified microRNA from the same PPP sample is provided in Fig. 1. In Additional file 1: Figure S1 Ct-values obtained by direct quantification of miR-92a, miR-16, and miR-126 in PPP from the 10 volunteers are presented with the corresponding results obtained using the conventional purification step. The approach was also tested using plasma as direct template for the reverse transcription, and since levels of miR-92a and miR-16 are higher in plasma compared to PPP [18], we expected the Ct-values to be lower when using plasma. However, no differences in Ct-values between the analysis using PPP and plasma were observed, which indicates that the RT-qPCR reaction was inhibited by components in the plasma (and maybe also in the PPP).

Finally, when using Approach number 4 Cel-miR-39 levels were undetermined in all samples. Ct-values for miR-92a were found to be 31–34 in plasma and 33–35 in PPP, which is in agreement with the fact that plasma contains a small number of platelets that will contribute to the microRNA pool [18]. In contrary, all Ct-values were > 35 for miR-126 and miR-16, regardless of the type of plasma used.

In all of the above outlined experiments a purified microRNA sample (from PPP) was included to serve as a positive control, and the Ct-values in these samples were consistently found to be between 21 and 30 depending on the microRNA measured.

Approach number 3, as inspired by Liu et al. [8], provided the lowest Ct-values of the four approaches tested. Still, especially for miR-16, the Ct-values were too high to provide reliable quantifications of the microRNA levels. Furthermore, the difference in Ct-value (ΔCt) between analysis performed directly with PPP and with miRNA purified from PPP were much higher for miR-16 (average ΔCt ≈ 16) compared to miR-92a (average ΔCt ≈ 11) and miR-126 (average ΔCt ≈ 8), Fig. 1 and Additional file 1: Figure S1.

Liu et al. [8] performed their analyses with serum and found for microRNA-126 Ct-values of 20–31, which is much lower than our results using plasma or PPP. To investigate whether the high Ct-values found in our experiments were due to inhibition of the RT-qPCR by components in the plasma, we performed additional analysis on prediluted (1:10 and 1:100) PPP and serum samples. Ct-values obtained for miR-126 and miR-16 in the undiluted samples were at least 35, and thus considered outside the measuring range. When diluting PPP or serum samples the Ct-values for miR-126 remained high, whereas Ct-values for miR-16 decreased, Table 2. This decrease in Ct-value with increasing sample dilution seen for miR-16 could indicate that an inhibition of the RT-qPCR occurred in the undiluted samples. When compared to the results published by Liu et al. [8], the high Ct-values found for miR-126 in our serum samples were surprising, but some differences exists between the two studies. First, whereas we used serum from tubes without gel separator and centrifuged the samples at 2000*g*, Liu et al. used serum from gel separator tubes, and after a centrifugation at 12,000*g* the samples were filtered through a serum filter. Secondly, we used the ABI Prism 7900HT whereas Liu et al. used a LightCycler system and thus another PCR master mix. For miR-92a the obtained Ct-values in some PPP samples were ~ 30, which is within a reasonable measuring range. Furthermore, when diluting the two PPP samples tenfold, we observed an increase in Ct-values of 4 and 2.9, respectively, Table 2. These values are within the expected range, as theoretically the Ct-value will increase by 3.3 when the microRNA levels decrease by tenfold. Nevertheless, when diluting the PPP samples by a 100-fold, we found no further increase in Ct-values, as compared to the tenfold dilution. Results obtained for miR-92a using serum samples were more inconsistent, Table 2.

**Table 2:** Ct-values obtained by approach number 3 using the buffer with pH 8

	miR-126		miR-16		miR-92a	
	Sample 1	Sample 2	Sample 1	Sample 2	Sample 1	Sample 2
PPP	38.4	35.9	37.1	35.0	30.5	29.5
PPP 1:10	39.7	39.1		34.0	34.5	32.4
PPP 1:100	38.5	38.3	32.5	29.8	34.7	32.7
Serum	37.9	40.9	37.0	40.6	31.5	37.6
Serum 1:10	40.1			37.8	33.4	36.9
Serum 1:100	38.0	38.4	32.6	35.0	32.5	34.8

The table provides Ct-values obtained in undiluted and diluted PPP and serum samples from two individuals. Empty cells represent undetermined values

The Ct-values obtained using the two denaturing buffers were similar, but more results were undetermined when using the buffer with the unadjusted pH.

Subsequently, to test whether components in the denaturing buffers inhibit/interferes with the RT-qPCR reactions, experiments on purified microRNA were performed in which 2.5 µL of denaturing buffer was added to the RT-reaction mixture. In addition, when purifying microRNA, we used 300 µL of PPP as starting material, and eluted the microRNA in 30 µL of water, so when performing the cDNA synthesis with 2 µL of purified microRNA sample, we add microRNA equivalent to 20 µL of PPP. Therefore, by performing the cDNA synthesis with 2.5 µL of purified microRNA prediluted 1:10 with water, we tested whether the 2.5 µL of PPP used in the cDNA synthesis in approach number 3 was sufficient to provide usable Ct-values. Similar Ct-values were obtained analyzing purified microRNA with and without addition of denaturing buffer which indicate that the buffers have no inhibitory effect (sample 1 and 2 in Additional file 2: Table S1). Furthermore, measurements with the diluted microRNA-sample were all within an acceptable measuring range (Ct-values between 26.2 and 31.3), and approximately 3 Ct higher than with the undiluted microRNA-sample, which is in agreement with the expected 3.3 Ct (sample 3 in Additional file 2: Table S1). These results indicate that we should have been able to obtain Ct-values within the measuring range when using 2.5 µL of PPP as template for the reverse transcription, and thus, that PCR inhibition occurred despite the small sample volume.

In conclusion, we did not achieve valid results by direct quantification of miR-92a, miR-16 or miR-126 in PPP using TaqMan assays without the microRNA-purification step.

## Limitations

It is a limitation of the study that we did not include analysis using the SYBR Green product specified in the cited references. However, at the time when we performed our investigations, the manufactures homepage was available in Chinese language only, and thus we were not able to acquire the exact same assays.

## Additional files

**Additional file 1: Figure S1.** Ct-values obtained using approach number 3. The plot shows the Ct-values for miR-16, miR-92a and miR-126 in samples from 10 volunteers. Results are obtained using platelet-poor plasma (PPP) direct as template for reverse transcription compared to analysis using miRNA purified from PPP (RNA).

**Additional file 2: Table S1.** Influence of buffer and test of sample volume. The table provides Ct-values obtained with three microRNA samples (purified from PPP). For sample 1 and 2 cDNA synthesis was performed with and without the addition of a denaturing buffer (with unadjusted pH or with pH adjusted to 8.0), and sample 3 was used undiluted and diluted 1:10 with water.

## Data Availability

Relevant data are included in the manuscript and the additional table.

## Abbreviations

PPP: platelet-poor plasma
RT-qPCR: reverse transcription quantitative real-time PCR
