# Supplementary material for: Quantification of microRNA in plasma using probe based TaqMan assays: is microRNA purification required?
Source: BMC Res Notes. 2019 May 10;12:261. doi: 10.1186/s13104-019-4301-5 (PMC6509816; doi:10.1186/s13104-019-4301-5)
Supplement: Supplementary file 1 — Additional file 1: Figure S1. Ct-values obtained using approach number 3. The plot shows the Ct-values for miR-16, miR-92a and miR-126 in samples from 10 volunteers. Results are obtained using platelet-poor plasma (PPP) direct as template for reverse transcription compared to analysis using miRNA purified from PPP (RNA). [file 13104_2019_4301_MOESM1_ESM.docx]

**Additional Figure S1:** Ct-values obtained using approach number 3


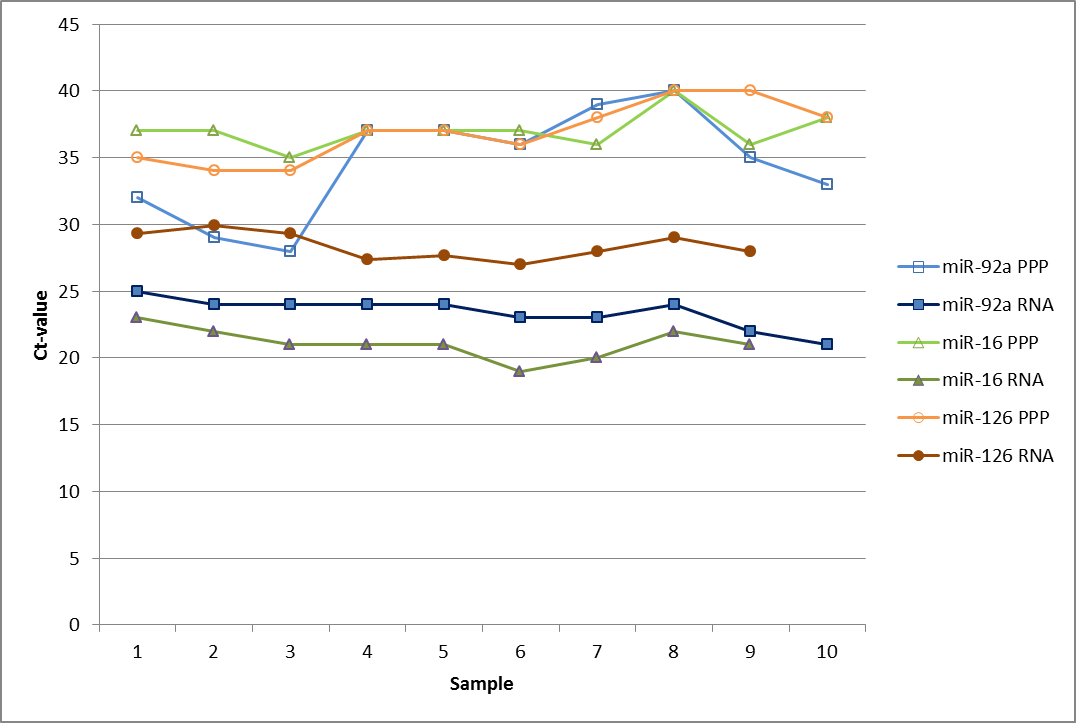


The plot shows the Ct-values for miR-16, miR-92a and miR-126 in samples from 10 volunteers. Results are obtained using platelet-poor plasma (PPP) direct as template for reverse transcription compared to analysis using miRNA purified from PPP (RNA).
