# Supplementary material for: Quantification of microRNA in plasma using probe based TaqMan assays: is microRNA purification required?
Source: BMC Res Notes. 2019 May 10;12:261. doi: 10.1186/s13104-019-4301-5 (PMC6509816; doi:10.1186/s13104-019-4301-5)
Supplement: Supplementary file 2 — Additional file 2: Table S1. Influence of buffer and test of sample volume. The table provides Ct-values obtained with three microRNA samples (purified from PPP). For sample 1 and 2 cDNA synthesis was performed with and without the addition of a denaturing buffer (with unadjusted pH or with pH adjusted to 8.0), and sample 3 was used undiluted and diluted 1:10 with water. [file 13104_2019_4301_MOESM2_ESM.docx]

**Additional Table S1: Influence of buffer and test of sample volume**

|  | **miR-126** | **miR-16** | **miR-92a** |
| --- | --- | --- | --- |
| Sample 1 | 29.2 | 21.4 | 24.7 |
| Sample 1 + buffer | 29.5 | 20.7 | 24.0 |
| Sample 1 + buffer (pH 8.0) | 29.7 | 20.9 | 24.1 |
| Sample 2 | 28.4 | 21.2 | 24.4 |
| Sample 2 + buffer | 28.4 | 19.8 | 23.0 |
| Sample 2 + buffer (pH 8.0) | 28.8 | 20.6 | 23.5 |
| Sample 3 | 28.4 | 23.2 | 25.2 |
| Sample 3 diluted 1:10 with H_2_O | 31.3 | 26.2 | 28.2 |
|  |  |  |  |

The table provides Ct-values obtained with three microRNA samples (purified from PPP).

For sample 1 and 2 cDNA synthesis was performed with and without the addition of a denaturing buffer (with unadjusted pH or with pH adjusted to 8.0), and sample 3 was used undiluted and diluted 1:10 with water.
